# Evaluation of intervention strategy of thalassemia for couples of childbearing ages in Centre of Southern China

**DOI:** 10.1002/jcla.23990

**Published:** 2021-09-07

**Authors:** Fan Jiang, Liandong Zuo, Jian Li, Guilan Chen, Xuewei Tang, Jianying Zhou, Yanxia Qu, Dongzhi Li, Can Liao

**Affiliations:** ^1^ Prenatal Diagnostic Center Guangzhou Women and Children’s Medical Center affiliated with Guangzhou Medical University Guangzhou China

**Keywords:** hematological parameters, hemoglobinopathy, intervention strategy, prevalence

## Abstract

**Background:**

To describe the free intervention strategy of thalassemia for childbearing couples in Guangzhou.

**Methods:**

Routine hematology examinations were conducted for 137,222 couples. Among them, 37,501 couples who had mean corpuscular volume (MCV) <82 fL or mean corpuscular hemoglobin <27 pg were elected for Hb analysis and the deletions of four common α‐thalassemia mutation. Reverse dot blot for common nondeletional α‐thalassemia and β‐thalassemia was selectively used. Three thousand twenty‐two couples randomly selected were offered all those tests as a control group. Sanger sequencing, multiplex ligation‐dependent probe amplification and next‐generation sequencing were used for rare thalassemia. High‐risk couples were offered prenatal diagnosis at 10–13 weeks’ gestation based on informed consent.

**Results:**

The carrier rates of α‐, β‐, and αβ‐thalassemia and δβ thalassemia/deletional HPFH were 7.7%, 3.02%, 0.5% and 0.059% respectively. Of them, 1.37% were identified as at‐risk couples and 345 couples terminated the pregnancy. No severe α‐ and β‐thalassemia births were observed. In the control group, two β‐ thalassemia carriers and one case with −α^3.7^/αα^QS^ were misdiagnosed, but all at‐risk couples were found, and we could save 1,523,774 ¥ using our strategy. The cut‐off points of 73.46 fL and 23.25 pg would be useful to find −α^+^/α^T^ thalassemia.

**Conclusion:**

The intervention strategy was cost‐effective and offered reference in population thalassemia screening.

## Graphical abstract

Flow‐chart for our thalassemia intervention strategy.

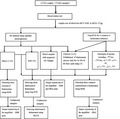

## 1 INTRODUCTION

Hemoglobinopathies are the most common Mendelian disorders including abnormal hemoglobin variants and thalassemia in the world.^1^ α thalassemia major leads to hydrops fetalis or perinatal death. β thalassemia major and some types of abnormal hemoglobin variants such as hemoglobin S, C compound with β thalassemia minor causes severe anemia. Carrier screening and high‐risk couple subjected to prenatal diagnosis are the main content of the thalassemia control plan.^2^, ^3^ Population‐screening programs for hemoglobinopathies have been performed in many countries in globe.^4^ The main impact factors for successful population screening programmes included political support, strong publicity in public, mutation spectrum of thalassemia in the local population, and cost effectiveness.^5^ Red blood cell indices including mean corpuscular volume (MCV) or mean corpuscular hemoglobin (MCH) and HbA2 quantification were often utilized to find carriers.^6^ Cut‐off values of hematologic parameters are used in determining whether molecular methods are needed,^7^ including Gap‐PCR and RDB for known breaking point or mutations and MLPA and next‐generation sequencing for rare genotypes of thalassemia.

In China, many places including Guangdong, Guangxi, Hailan, Fujian, Taiwan and Hong Kong performed the carrier screening for thalassemia.^8^, ^9^, ^10^, ^11^, ^12^, ^13^ Except Hongkong and Taiwan, the objectives of carrier screening in other places were focused in the molecular spectrum of thalassaemia.^14^ Our center established the hospital‐based program and proved to be highly effective in reducing severe thalassemia in pregnant populations,^15^ but only few genotypes were included and the program was not detailed. As the centra of South China, a large number of migrants have settled in recent years and migrations may change the mutation spectrum. Pre Gestational Thalassemia Screening program has been carried out for 4 years. The objective of our study is to evaluate the current population screening strategy based on government support and multiple departments included, which can provide evidence for establishing the guideline of population thalassemia screening.

## 2 MATERIALS AND METHODS

### 2.1 Ethical study subjects and prenatal screening strategy

**FIGURE 1:**
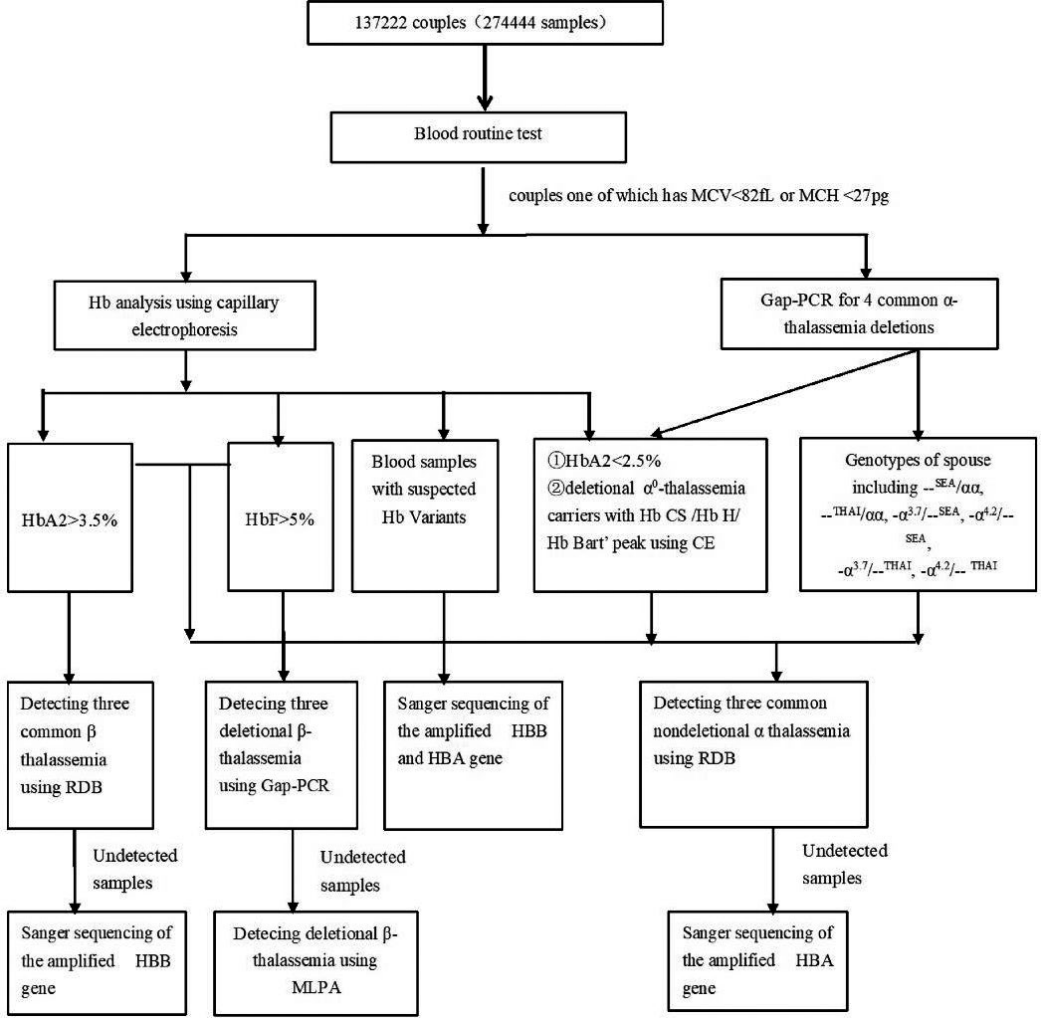
The intervention strategy of thalassemia for couples of childbearing age in Guangzhou

Pre Gestational Thalassemia Screening Program was performed through the three‐level network of maternal and child health in Guangzhou area and supported by the government. From 2016 to 2019, 274,444 cases (137,222 couples) from twelve districts took part in total. They all belonged to the household registered population and signed the informed consent in local primary healthcare centers. Pre‐test counselling, routine hematology examinations were carried out in basic Maternal and Child Health Care Institution. Internal quality control and external quality assessment (EQC) were performed to ensure precision and consistency of results. From the 37,501 couples, 74,102 subjects who had MCV < 82 fL or MCH < 27 pg were transported to our center, which belongs to the third‐class maternity children hospital for further investigation. All of them were performed Hb analysis using capillary electrophoresis (CE; Serbia, Paris, France). Four common α‐thalassemia deletions were also tested using gap‐PCR in those samples, including ‐‐^SEA^, ‐α^3.7^, ‐α^4.2^ and ‐‐^Thailand^. Molecular analysis for common nonrecreational α‐thalassemia was performed according to the following conditions (1) the samples without four common deletional α thalassemia but positive for thalassemia screening (MCV < 82 fL, MCH < 27 pg or Hb A2 < 2.5%); (2) the partner with ‐‐^SEA^/αα, ‐‐^Thailand^ /αα or Hb H disease; (3) the cases with CS peak observed on electrophoresis; (4) the samples with Hb H or Hb Bart's but negative for common deletional α thalassemia detection; and (5) β‐thalassemia carriers. Samples with HbA2 > 3.5% or HbF > 5% were detected by RDB to eliminate seventeen common mutations in the HBB gene. For cases with HbF levels ≥5%, we used Gap‐PCR to screen three types of δβ‐thalassemia/deletional HPFH reported in some studies in China, including Chinese^G^γ(^A^γδβ)^0^‐thalassemia, SEA‐HPFH and Taiwanese deletion. Two thalassemia gene detection kits were used to perform these assays (Shenzhen Yishengtang Biological Products Co., Ltd.). Sanger sequencing was carried out for samples with abnormal variants or negative RDB results for common mutations of β‐globin gene. Gap‐PCR was used for cases with HbA2 levels ≥3.5% but negative for sanger sequencing or Hb F levels ≥5% to screen three common δβ‐thal/deletional HPFH. MLPA was used to investigate whether uncommon delusional α or β thalassemia existed. Next‐generation sequencing was used to find deletional point or the subjects with suspected family history of thalassemias. The strategy was presented in Figure 1.

### 2.2 Molecular analysis for the control group

About 7044 samples from 3522 couples were randomly chosen as the control group. All samples received Hb analysis and common molecular analysis including four common deletional α‐thalassemia, three common nondeletional α‐thalassemia and seventeen common mutation β‐thl.

### 2.3 Statistical analysis

Nonparametric Mann–Whitney *U* test was conducted by analyzing the hematological parameters differences between groups. The difference of carrier rate of hemoglobinopathies in four types of districts was conducted by Pearson Chi‐square analysis. A *p*‐value of less than 0.05 was regarded as a significant difference. ROC (the receiver operator characteristic) was performed to determine the cut‐off value of Hb E, MCH and MCV. All those data were analyzed using SPSS V.19.0 software.

## 3 RESULTS

### 3.1 Screening status in Guangzhou

**FIGURE 2:**
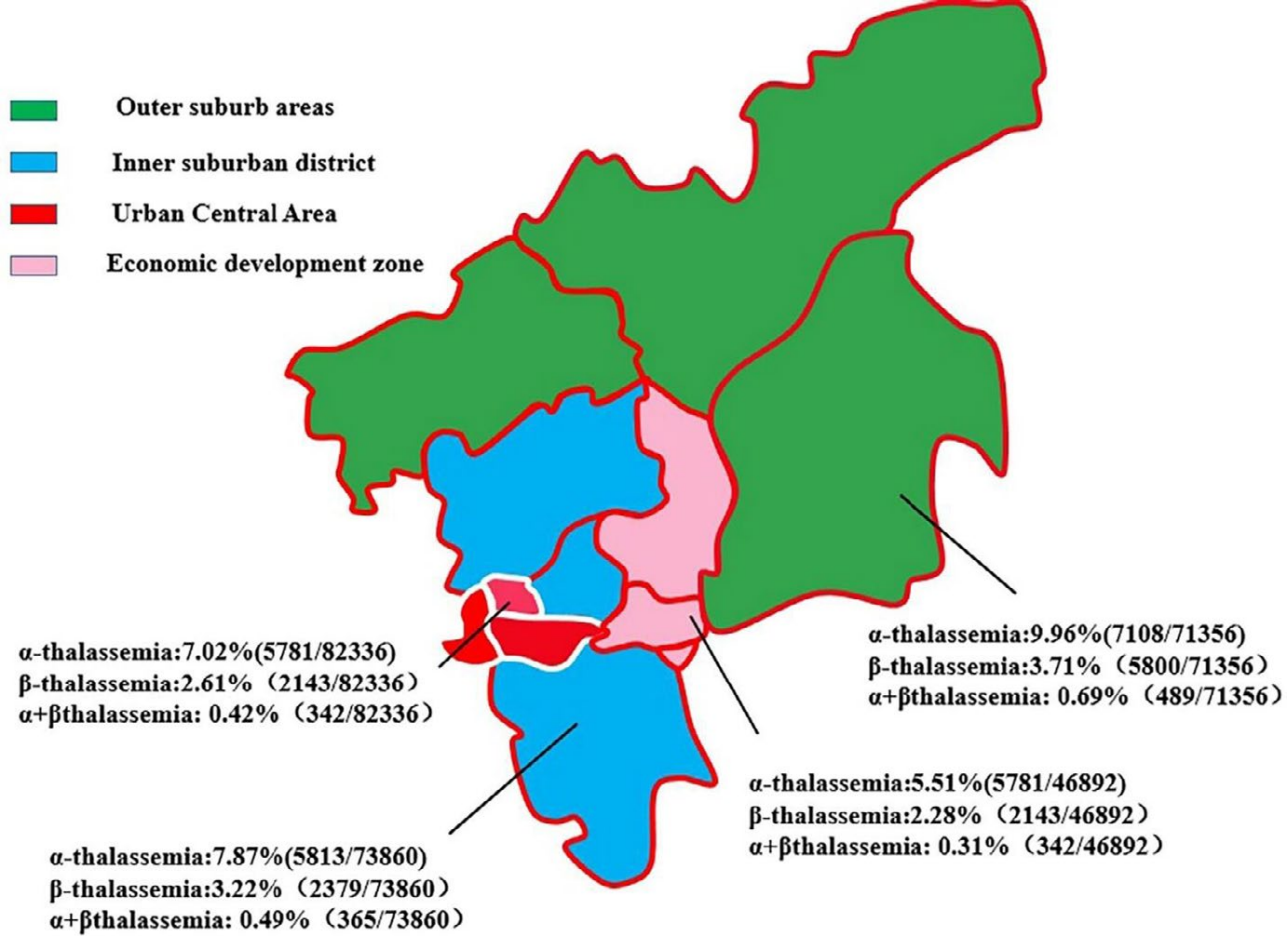
The carrier rate of thalassemia in four different types of districts in Guangzhou

The overall carrier rate of thalassemia was 11.34%, including 7.76% for α‐thalassemia, 3.02% for β‐thalassemia and 0.49% for combined α‐/β‐thalassemia. The carrier rate of δβ/delusional thalassemia was about 0.059%. 0.69% of couples had high risk of delivering a baby with thalassemia major and received genetic counselling. Three hundred and forty‐five couples chose to terminate the pregnancy due to affected fetuses. No infants were born with thalassemia major in this screening program. According to the distance from the city center and economic development level, eleven districts in Guangzhou area can be divided into four types: the outer suburb areas, the inner suburban district, the central city district and the economic development zone. The carrier rates of α‐thalassemia, β‐thalassemia and combined α‐/β‐thalassemia were highest in the outer suburb areas while it was lowest in the economic development zone (Figure 2).

### 3.2 Gene mutation spectrum and hemorheology characteristics of thalassemia

Molecular spectrum of persons with α, β and αβ thalassemia were shown separately as a top‐Fifteen list in Table 1. Hematology parameters of them were shown in Table 2. Thirty‐two genotypes of α thalassemia, thirty‐five genotypes of β thalassemia and seventy‐two genotypes of αβ thalassemia were identified in our study. Specifically, the most frequent mutation of β thalassemia −28(A>G) in Nansha District belonging to the economic development zone which was very distinctive from other districts. Beyond that, Ninety‐five carriers with Chinese ^G^γ(^A^γδβ)^0^‐thalassemia, 53 cases with SEA‐HPFH, seven cases with Taiwanese deletion have been diagnosed by gap‐PCR. We also identified two cases of δβ thalassemia. One of them was initially reported by our team before while the δβ thalassemia was never described (Figure S1). α^WS^α/αα rather than α^QS^α/αα coexisted with β^CD41‐42(‐CTTT)^/β^N^ was the most common genotype in carriers with concurrent nontraditional α‐ and β‐thalassemia. Considering the correlation between parameters and genotypes, we found one case with rare α thalassemia which was described in Figure S2. Twenty‐five different types of hemoglobinopathies were found in our study (Table S1).

**TABLE 1:** Molecular spectrum of persons with thalassemia (Number ≥2)

Genotype (α‐thal) thalassemia)	Numbers	%	Genotype (β‐thal)	Numbers	%	Genotype (αβ‐thal)	Numbers	%
‐‐^SEA^/αα	13715	64.44	β^CD41‐42(‐CTTT)^/β^N^	3261	39.58	‐‐^SEA^/αα, β^CD41‐42(‐CTTT)^/β^N^		19.71
‐α^3.7^/αα	3742	17.58	β^IVS2‐654(C>T)^/β^N^	2146	26.04	‐‐^SEA^/αα, β^IVS2‐654(C>T)/^β^N^	213	15.89
‐α^4.2^/αα	1636	7.69	β^‐28(A>G)^/β^N^	1501	18.22	‐‐^SEA^/αα, β^‐28(A>G)/^β^N^	133	9.93
α^QS^α/αα	677	3.18	β^CD17(A>T)^/β^N^	748	9.08	‐α^3.7^/αα, β^CD41‐42(‐CTTT)^/β^N^	109	8.13
α^WS^α/αα	514	2.41	β^CD71‐72(+A)^/β^N^	163	1.98	‐α^3.7^/αα, β^IVS2‐654(C>T)/^β^N^	92	6.87
α^CS^α/αα	411	1.93	β‐^29(A>G)^/β^N^	87	1.06	‐α^4.2^/αα, β^CD41‐42(‐CTTT)^/β^N^	55	4.10
‐‐ ^SEA^/‐α^3.7^	205	0.96	β^CD43(G>T)^/β^N^	80	0.97	‐α^3.7^/αα, β^−28(A>G)/^β^N^	54	4.93
‐‐ ^SEA^/‐α^4.2^	104	0.49	β^CD27‐28(+C)^/β^N^	66	0.80	‐‐^SEA^/αα, β^CD17(A>T)^/ β^N^	44	3.28
‐‐^THAI^/αα	59	0.28	β^IVS1‐1(G>T)^/β^N^	65	0.79	‐α^3.7^/αα, β^CD17(A>T)^/ β^N^	37	2.76
‐α^3.7^/‐α^4.2^	55	0.26	β^CD14‐15(+G)^/β^N^	35	0.42	α^WS^α/αα, β^CD41‐42(‐CTTT)^/β^N^	36	2.84
‐α^3.7^/‐α^3.7^	52	0.24	β^‐90(C>T)^/β^N^	18	0.22	‐α^4.2^/αα, β^IVS2‐654(C>T)/^β^N^	28	2.09
‐‐ ^SEA^/HKαα	16	0.08	β^CD37(TGG>TAG)^/β^N^	16	0.19	‐α^4.2^/αα, β^‐28(A>G)/^β^N^	26	1.94
‐α^3.7^/αα^CS^	13	0.06	β^‐88(C>T)^/β^N^	9	0.11	α^WS^α/αα, β^IVS2‐654(C>T)/^β^N^	26	1.94
‐α^4.2^/‐α^4.2^	12	0.06	β^INT(T>G)^/β^N^	8	0.10	‐‐^SEA^/αα, β^IVS1‐1(G>T)^/ β^N^	11	0.82
‐α^3.7^/αα^QS^	12	0.06	β^CD89‐93 (‐14bp)^/β^N^	6	0.07	‐α^3.7^/αα, β^CD71‐72(+A)^/ β^N^	11	0.82

**TABLE 2:** Hematological parameters of persons with thalassemia (Number ≥ 2)

Genotype	Hb (g/L)	MCV (fL)	MCH (pg)	HbA2 (%)
*α thalassemia*				
Silent α‐carrier	135.34 ± 15.54	81.16 ± 4.62	26.13 ± 1.74	2.53 ± 0.27
‐α/αα (‐α^3.7^, ‐α^4.2^)	137.17 ± 14.21	82.37 ± 3.51	26.61 ± 1.65	2.55 ± 0.21
^a^α^T^/αα				
α^QS^α/αα	133.28 ± 13.02	75.53 ± 3.77	24.00 ± 1.24	2.56 ± 0.23
α^WS^α/αα	136.82 ± 16.58	82.04 ± 4.54	26.56 ± 1.81	2.69 ± 0.25
α^CS^α/αα	132.50 ± 15.85	80.65 ± 4.39	25.98 ± 1.66	2.17 ± 0.22
HKαα/α^4.2^	142.0 ± 16.90	81.63 ± 5.26	25.84 ± 1.6	2.55 ± 0.07
α thalassemia minor				
‐‐/αα(‐‐^SEA^, ‐‐^THAI^)	127.71 ± 13.31	68.21 ± 3.46	21.39 ± 1.05	2.31 ± 0.17
‐α/‐α(‐α^3.7^/‐α^4.2^, ‐α^3.7^/‐α^3.7^, ‐α^4.2^/‐α^4.2^)	127.09 ± 13.91	72.26 ± 3.21	22.56 ± 1.14	2.42 ± 0.19
‐α/α^T^(‐α^3.7^/αα^QS^, ‐α^3.7^/αα^WS^, ‐α^4.2^/αα^CS^, ‐α^4.2^/αα^QS^,‐‐^THAI^/α^QS^, ‐α^4.2^/αα^WS^)	121.33 ± 12.61	71.28 ± 5.47	22.05 ± 1.86	2.25 ± 0.39
α^T^/α^T^(αα^WS^/αα^WS^, αα^WS^/αα^CS^)	124.83 ± 15.74	75.66 ± 6.75	23.96 ± 2.64	2.49 ± 0.44
^a^‐‐^SEA^/HKαα	127.7 ± 13.86	66.63 ± 4.01	20.61 ± 1.27	2.43 ± 0.84
^a^‐‐^SEA^/αα^WS^	117.42 ± 12.79	62.99 ± 5.95	20.67 ± 1.82	2.79 ± 1.26
Hb H disease				
‐‐/‐α(‐‐^SEA^/‐α^3.7^, ‐‐^SEA^/‐α^4.2^, ‐‐^THAI^/‐α^3.7^)	98.23 ± 12.41	60.17 ± 5.44	17.86 ± 1.30	1.10 ± 0.29
‐α/‐αα^T^(‐‐^SEA^/αα^CS^, ‐‐^SEA^/αα^QS^)	91.38 ± 12.96	72.93 ± 8.19	19.18 ± 1.43	0.63 ± 0.20
*β thalassemia*				
^a^Silent β‐carrier	123.12 ± 13.99	66.61 ± 5.51	21.13 ± 1.82	5.33±0.46
CD26(G>A)	133.16 ± 14.52	76.76 ± 3.36	25.18 ± 1.22	3.65 ± 0.35
‐28(A>G),‐29(A>G),90(C>T),‐88(C>T)	128.67 ± 13.17	71.38 ± 3.76	22.73 ± 1.24	5.62 ± 0.41
IVS2‐654(C>T)	119.09 ± 13.16	63.08 ± 3.64	19.95 ± 1.64	5.12 ± 0.37
β thalassemia minor				
CD41‐42(‐CTTT), CD17(A>T),CD71‐72(+A), CD43(G>T), CD27‐28(+C),IVS1‐1(G>T), CD14‐15(+G), CD37(TGG>TAG), INT(T>G), CD89‐93 (‐14bp), IVS1‐5(G>C), IVS‐II‐705 (T>G), CD54‐58(‐13 bp), IVS‐II‐2(‐T), IVS‐I‐2 (T>A)	118.78 ± 13.09	62.80 ± 3.48	19.86 ± 1.16	5.46 ± 0.411
^b^β thalassemia intermedia	106.8 ± 21.6	65.73 ± 1.39	21.08 ± 1.05	4.56 ± 1.17
αβ thalassemia				
Concurrent α^+^ and β^+^ thalassemia	128.60 ± 14.81	69.71 ± 6.48	22.13 ± 2.35	5.13 ± 0.38
Concurrent α^+^ and β^0^ thalassemia	123.62 ± 13.64	65.40 ± 3.91	20.65 ± 1.24	5.52 ± 0.42
Concurrent α^0^ and β^+^ thalassemia	129.18 ± 14.16	70.79 ± 4.12	22.47 ± 1.52	5.14 ± 1.38
Concurrent α^0^ and β^0^ thalassemia	127.15 ± 14.61	70.41 ± 4.36	22.50 ± 1.52	5.38 ± 0.41
Concurrent Hb H disease and β^+^ thalassemia	126.58 ± 13.29	52.8 ± 3.84	16.88 ± 0.88	4.48 ± 0.46
Concurrent Hb H disease and β^0^ thalassemia	113.05 ± 11.7	52.13 ± 2.57	16.75 ± 0.70	4.72 ± 0.19
Concurrent HbE and α^+^ thalassemia	138.16 ± 10.86	77.99 ± 4.25	25.41 ± 1.01	3.91 ± 0.32
Concurrent HbE and α^0^ thalassemia	125.46 ± 10.78	69.69 ± 3.36	21.90 ± 0.94	3.82 ± 0.26

^a^ Hematological parameters and genotypes were listed when there were significant differences in MCV and MCH values among genotypes within the group; Hematological parameters of rare genotypes were listed.

^b^ The β thalassemia intermedia carriers have HbF>20% which were not listed in this table.

### 3.3 Red blood cell indices and hemoglobin analysis using in this strategy

**FIGURE 3:**
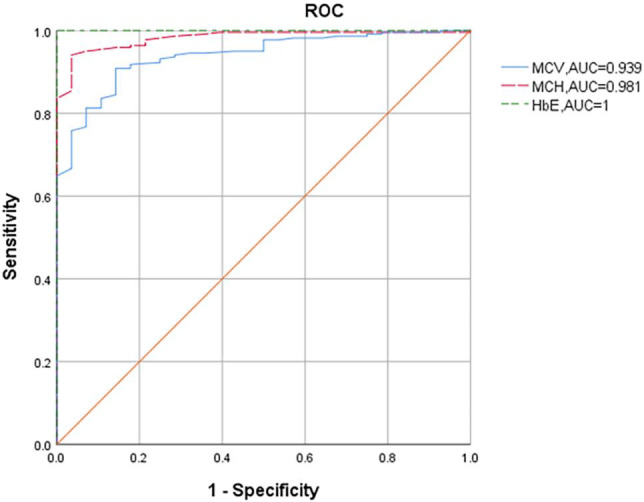
(a) ROC curve analysis for MCV, MCH and HbA2 in distinguishing α^+^‐thalassemia carriers and α0‐thalassemia carriers

Among the 32985 samples with MCV values >82 fL and MCH values >27 pg in our study, α^+^ thalassemia carriers were common, including ‐α^3.7^/αα (2.05%) and ‐α^4.2^/αα carriers (0.66%). 8.27% of α^CS^/αα (35/411) and 16.92% of α^WS^/αα carriers were also observed to have normal MCV and MCH values. None of cases with α^QS^/αα had normal MCV and MCH values. Receiver operating characteristic (ROC) curve showed that MCV and MCH could be efficient to distinguish between α^+^ thalassemia and α^0^ thalassemia carriers (the AUC of 0.978 and 0.977 respectively; the cut‐off point of 73.46 fL and 23.25 pg respectively; Figure 3). Only three of β thalassemia carriers had MCV values >82 fL and MCH values >27 pg at the same time. The genotype of those people was β^−28(A>G)^/β^N^. Most carriers with Hb variants have normal MCV and MCH values except for Hb E. Only two carriers with Hb E had normal MCV and MCH levels.

The 97.8% of samples with HbA2 > 3.5% have been found to have a HBB gene mutation. Hb A2 level of five individuals with β thalassemia and one case with αβ‐ thalassemia were lower than 3.5%, including two cases with β^IVS‐Ⅱ‐654 (C>T)^ /β^N^, two cases with β^CD41‐42(‐TTCT)^/β^N^, one cases with β^CD27‐28(+C)^/β^N^ and one case with β‐^28(A>G)^/β^N^ coexisted with ‐‐^SEA^/αα. All those cases have higher Hb F so that we could identify them. A δ gene mutation (HBD:c.‐127T>C) has been identified in four cases. All those individuals with Hb variants got genetic testing for β thalassemia. Hb A2 levels of persons with Hb variants coexisted with β thalassemia were also more than 3.5%, but abnormal Hb levels were affected. Fifty‐four cases with HbE had Hb A2 level lower than 3.5%. Hb E level is a useful indicator, especially discriminating HbE/α^0^ thalassemia from HbE heterozygotes. The appropriate cutoff values for founding HbE/α^0^ thalassemia were an Hb E level of less than 19.4% by CE, a MCV of less than 72.27 fL and a mean corpuscular hemoglobin (MCH) level of less than 23.35 pg (Figure S3). Hb E level of Hb E/β^+^ thalassemia carriers was higher than 40% while Hb level was more than 90 g/L.

### 3.4 The result of molecular analysis of the control group

All those samples were detected common mutations in the HBB gene and common nondeletional/deletional α thalassemia. We compared the results and cost using our screening strategy. Using our strategy, 2679 cases and 957 cases need to be tested using the polymerase chain reaction (PCR)‐reverse dot‐blot assay for nondeletional α‐thalassemia and β‐thalassemia respectively. According to charge for the kit (the average cost of β‐thalassemia genetic test is 158.15¥; the average cost of nondeletional α‐thalassemia genetic test is 129.17¥), we could save 1,526,486¥. The misdiagnosis cases included two β thalassemia carriers, five cases with αα^WS^/αα^WS^, two cases with ‐α^4.2^/αα^QS^, two cases with ‐α^3.7^/αα^WS^, one case with ‐α^3.7^/αα^QS^ and one case with ‐α^4.2^/αα^WS^. Those two β thalassemia carriers were not detected because their Hb A2 levels were less than 3.5% and Hb F were less than 5%. One case was identified to have a δ gene mutation (HBD:c.‐127T>C). The genotype of another case was ‐‐^SEA^/αα combined with β^N^/β^CAP40‐43 (‐AAAC)^. If we used our strategy and the cut‐off point of 73.46 fL and 23.25 pg as one of selection criteria for discriminating –α/α^T^ thalassemia and α^+^ thalassemia carriers, only one case with –α^3.7^/αα^QS^ would be undiagnosed but we could save 1,523,774¥. No at‐risk couples were misdiagnosed.

## 4 DISCUSSION

As the center of Guangdong province, progestational thalassemia screening has been performed in Guangzhou to reduce the birth rate of thalassemia major. Comparing to other population screening in China, the government leading and multi‐sectors participating for this program ensured the largest number of participants. Most importantly, the three‐level healthcare network played an important part in this work. The stuff in local healthcare centers performed education, pre‐test counselling, blood routine tests, interpretation of results and follow‐up for families not being at risk of having children with severe thalassemia. Additional examination, prenatal diagnosis and quality control were performed in our center. The follow‐up results showed that no severe α‐ and β‐thalassemia births were observed in participants.

According to references and our economic situation, we chose cut‐off of 82 fL for MCV and 27 pg for MCH as the primary screening index to identify couples at risk.^16^ Although α^+^‐thalassemia carriers would be misdiagnosed using the cut‐off values, the couples at risk of having a baby with deletional HbH could be detected because all of couples one of which has the lower MCV or MCH got genetic testing for four common deletional α‐thalassemias. Most of the couples at risk of having a baby with nondeletional HbH could be identified using our strategy. The cases with Hb CS homozygosity have been reported to present with fetal anemia and hydrops before.^17^ In our study, 8.27% of the α^CS^α/αα carriers (35/411) had MCV values >82 fL and MCH > 27 pg, which meant that the couples at risk of having a baby with α^CS^/α^CS^ would be misdiagnosed if all of them had normal MCV and MCH values. The prevalence of Hb CS was reported 0.3% in South China by our team.^18^ Based on the above data, only 0.0006% of couples may be at‐risk of having children with α^CS^/α^CS^. The rate of missed diagnosis was very low. In addition, we also find three people with ‐‐^SEA^/αα had MCH values >26 pg. Alpha globin cluster duplications were observed in those cases. Beta thalassemia intermedia due to coexistence of β thalassemia and alpha globin cluster duplications has been reported before.^19^

A combined MCV and MCH for screening β‐thalassemia was also recommended.^20^ In our study, 0.015% of 32985 cases with normal MCV and MCH were detected to be β‐thalassemia carriers or Hb E heterozygotes. In the control group, no β‐thalassemia carriers were observed to have normal MCV and MCH. The data showed that the rate of missed diagnosis would be very low. HbA2 > 3.5% is diagnostic of β‐thalassemia. Accounting for 0.06% of β‐thalassemia carriers and 0.01% of αβ‐thalassemia carriers had HbA2 level less than 3.5%. All those cases were diagnosed owing to Hb F of them more than 5%. Although Hb F was not the excellent parameter for screening β thalassemia coexisted with HBD mutation, we should pay more attention to those cases with elevated Hb F > 5% especially their spouses were also β thalassemia carriers. We found only one case with β^+^ thalassemia composite α^+^ thalassemia having normal Hb A2 level. The genotype for him was ‐‐^SEA^/αα compounded with β^N^/β^CAP40−43 (–AAAC)^ Three cases with β^+^/β^CAP40‐43 (–AAAC)^ or β^0^/β^CAP40‐43 (–AAAC)^ presented mild anemia and one case with β^CAP40‐43 (–AAAC)^/β^N^ presented normal in our study. We inferred that this mutation may not cause β‐thalassemia. It needs us do more research. Our study showed that 2.3% of the high‐risk fetus for beta‐thalassemia major also had high risk for hydrops fetalis at the same time. It is very essential to screen α‐thalassemia in β‐thalassemia carriers when the spouse was α‐thalassemia carrier. The Hb E level of less than 19.4% could be as the best cut‐off value for discriminating Hb E heterozygotes and Hb E.

Hb F > 5% would be useful for detecting δβ‐thalassemia and HPFH, which could also cause beta thalassemia intermedia compounded with β thalassemia. Two carriers with rare β‐globin gene cluster deletion were also identified in those people. One person with rare (^A^γδβ)^0^ thalassemia which was first reported by our laboratory in 2016. δβ thalassemia was common in cases with high Hb F and low HbA2. MLPA was so beneficial for us to identify δβ thalassemia carriers and NGS could provide the correct basis to breakpoint which was helpful for prenatal diagnosis.

Although the carriers with common Hb variants usually have normal MCV and MCH level except for Hb E and Hb Q‐Thailand carriers, Hb variants compounded with thalassemia will not cause severe anaemia. In our study, all those cases with Hb variants were detected common genotypes of thalassemia. The Hb A2 level was more than 3.5%, and MCV and MCH levels were lower than cut‐off value in persons with Hb variants compounded with β thalassemia. It also identified that the screening values of MCV and MCH in population screening.

Using the strategy, we also described the carrier rates of α‐thalassemia, β‐thalassemia and combined α‐/β‐thalassemia in Guanghzou. The carrier rates were all lower than those identified in Guangdong province by Aihua Yin^21^ (11.34% vs. 17.83%, 7.76% vs. 12.96%, 3.02% vs. 3.98% and 0.49% vs. 0.64%, respectively). It also revealed the significant discrimination between different areas in Guangzhou area. The carrier rate was the highest in the outer suburb which meant that the government should pay greater attention to thalassemia screening in those areas. The gene mutation spectrum was similar to data in Guangdong province reported by Xu and Aihua Yin.^22^ The slight difference between them including the constituent ratio for nondeletional α‐thalassemia and β‐thalassemia. The detection rate of α^QS^α/αα was highest while that of α^WS^α/αα was highest in Guangdong province. Interestingly, we found that α^WS^ compound with CD41‐42(‐CTTT) was the most frequent genotype of αβ‐thalassemia. All αα^QS^/αα carriers had MCV < 82 fL and MCH < 27pg while most α^WS^/αα carriers had normal MCV and MCH which may cause the latter to be undiagnosed. Based on those data, we speculated that α^WS^α/αα was also the most common nondeletional α‐thalassemia. The proportion of migrant population is highest (74.6%) in Nansha District may cause −28(A>G) was the most frequent HBB gene mutation in this place. All those data were further demonstrated that the genotypes of β‐thalassemia are very different owing to population constitution.

This is the first time to provide a detailed description for population screening strategy based on the government leading and multi‐sectors participating. The values of MCV, MCH, Hb A2 and Hb F of partners, multi methods combined for genetic testing and follow‐up for families being at‐risk are very useful in large population screening. The biggest data in our study could provide laboratory support to genetic counselling and intervention strategy of thalassemia.

## CONFLICT OF INTERESTS

The authors in our study do not have any conflict of interest, financial or otherwise.

## AUTHOR CONTRIBUTIONS

Can Liao and Dongzhi Li contributed the central idea, Fan Jiang analyzed most of the data and wrote the initial draft of the paper, Duolian Zuo and Jian Li were responsible for project management, Molecular experiments were performed by Xuewei Tang and Guilan Chen. Jianying Zhou and Yanxia Qu were responsible for CE and data analysis. All authors reviewed the results and revised the manuscript.

## Supporting information

Fig S1‐S3

Table S1

## Data Availability

All the data generated and/or analyzed in this work are available from the corresponding author according to request (canliao7981@21cn.com).
