# Supplementary material for: Evaluation of intervention strategy of thalassemia for couples of childbearing ages in Centre of Southern China
Source: J Clin Lab Anal. 2021 Sep 7;35(10):e23990. doi: 10.1002/jcla.23990 (PMC8529143; doi:10.1002/jcla.23990)
Supplement: Supplementary file 1 — Fig S1‐S3 [file JCLA-35-e23990-s001.docx]

**F I G U R E S1**


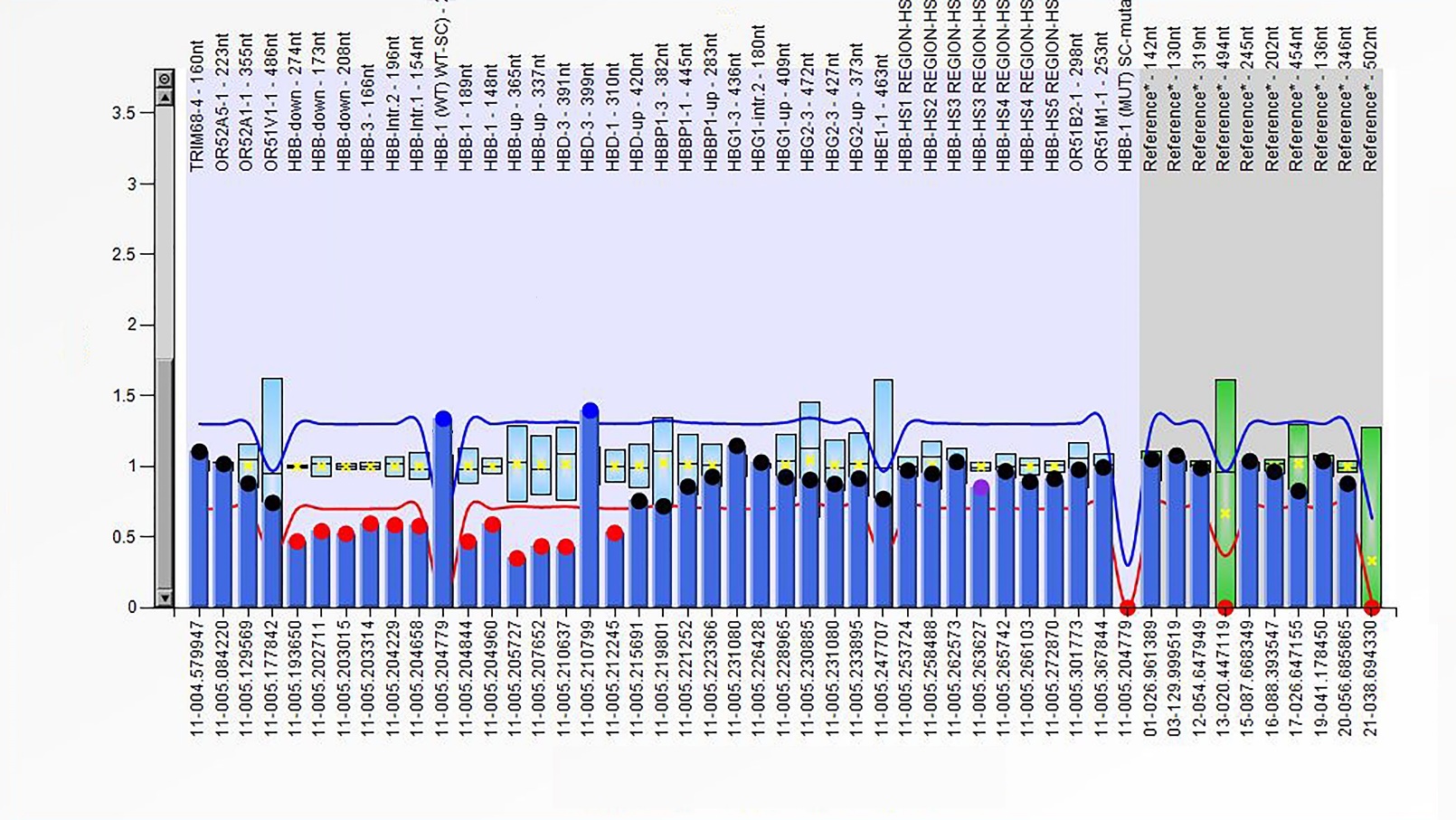


**(a)**


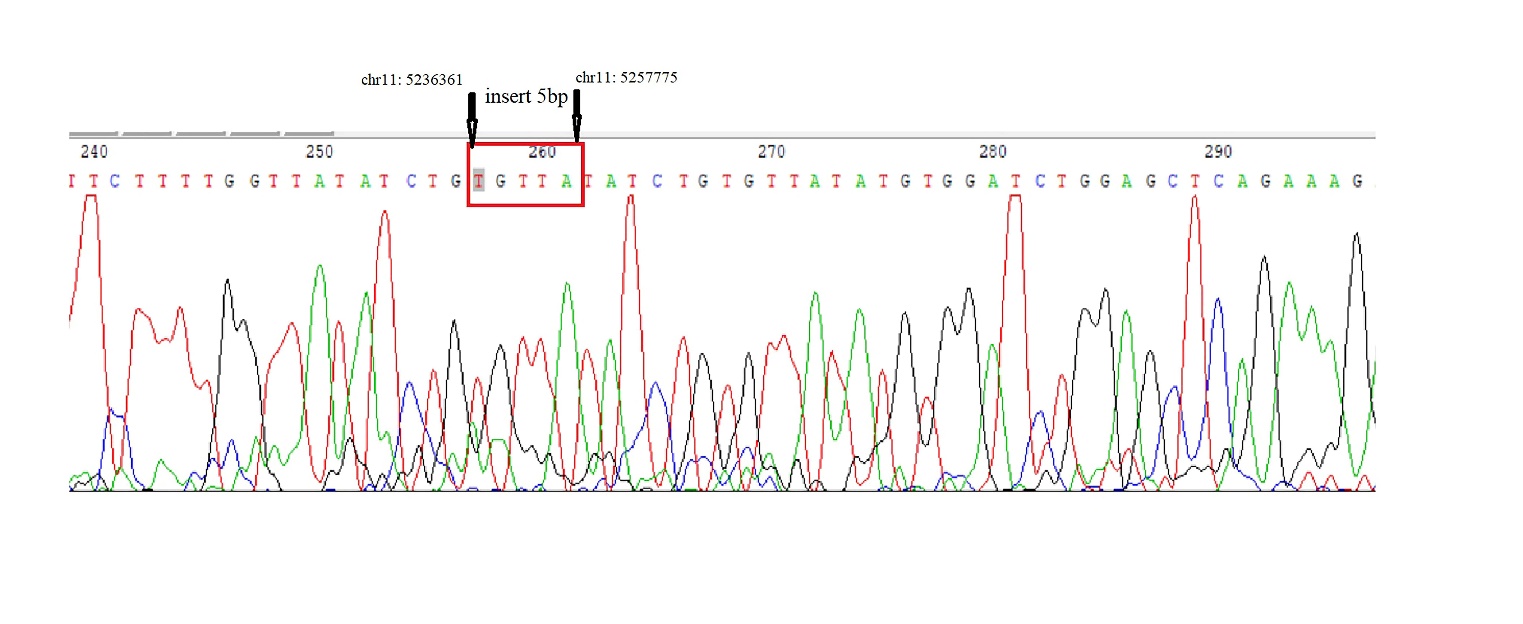


**(b)**

**F I G U R E S2**


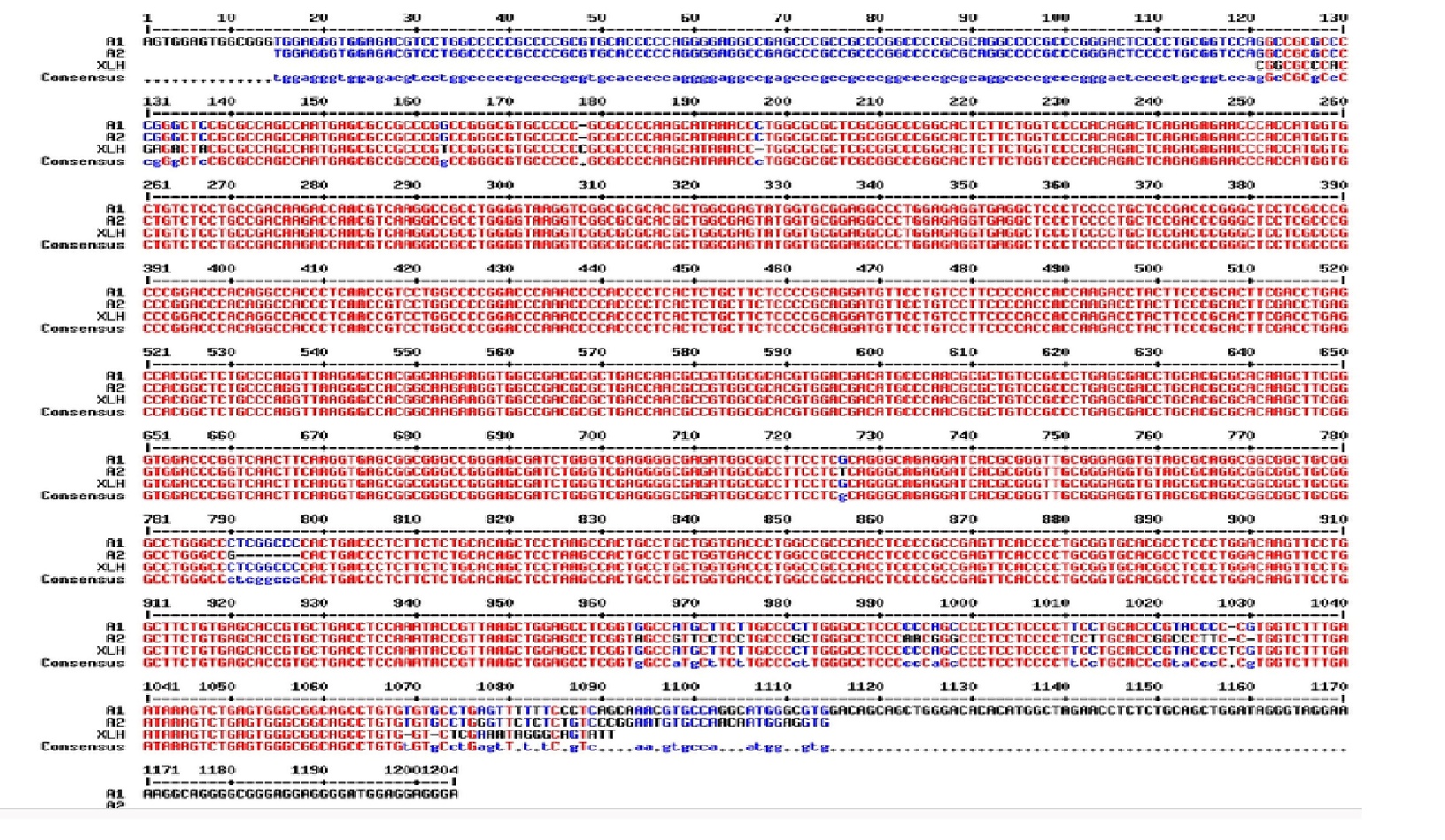


**(a)**


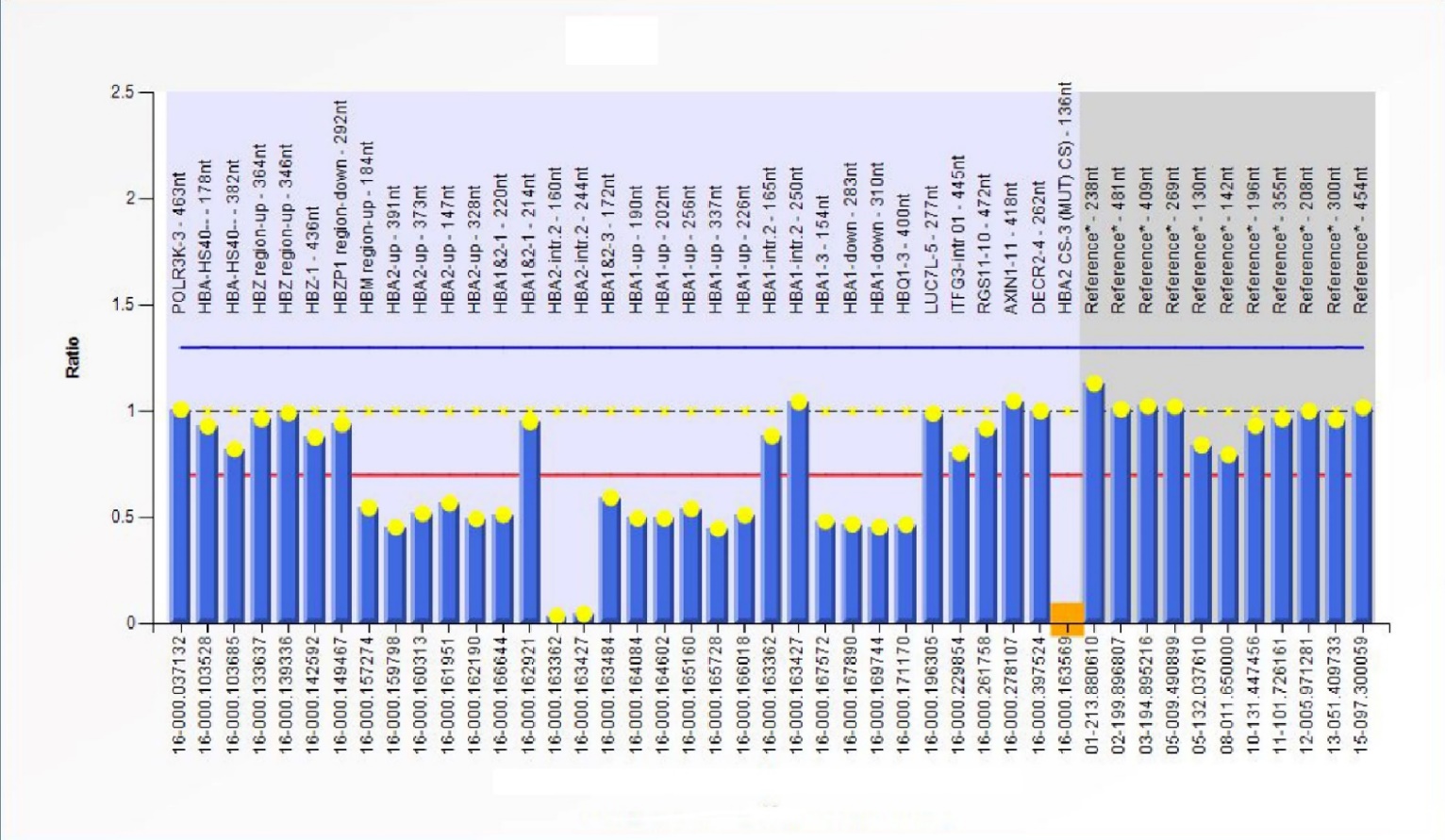


**(b)**


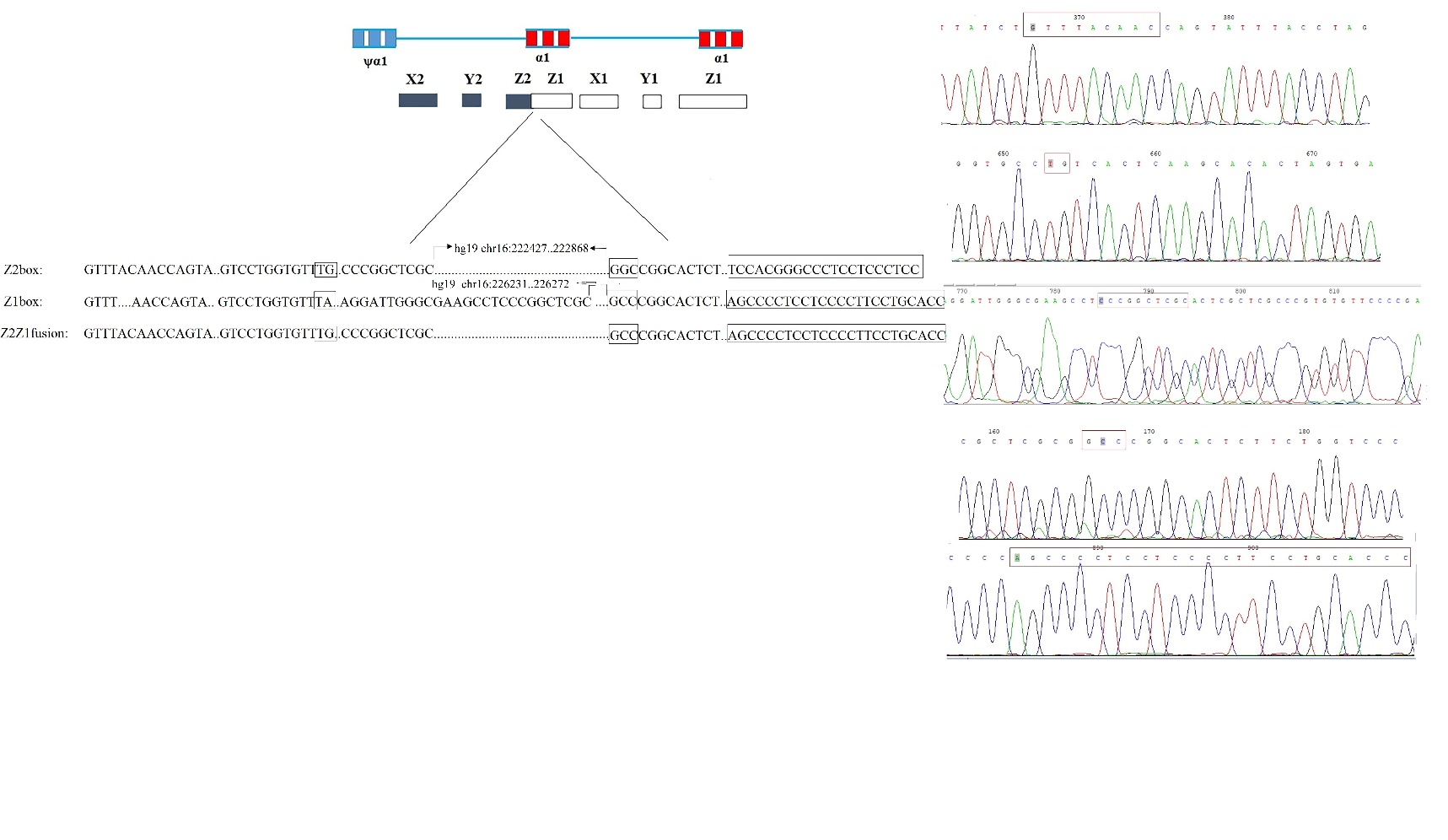


**(c)**

**F I G U R E S3**


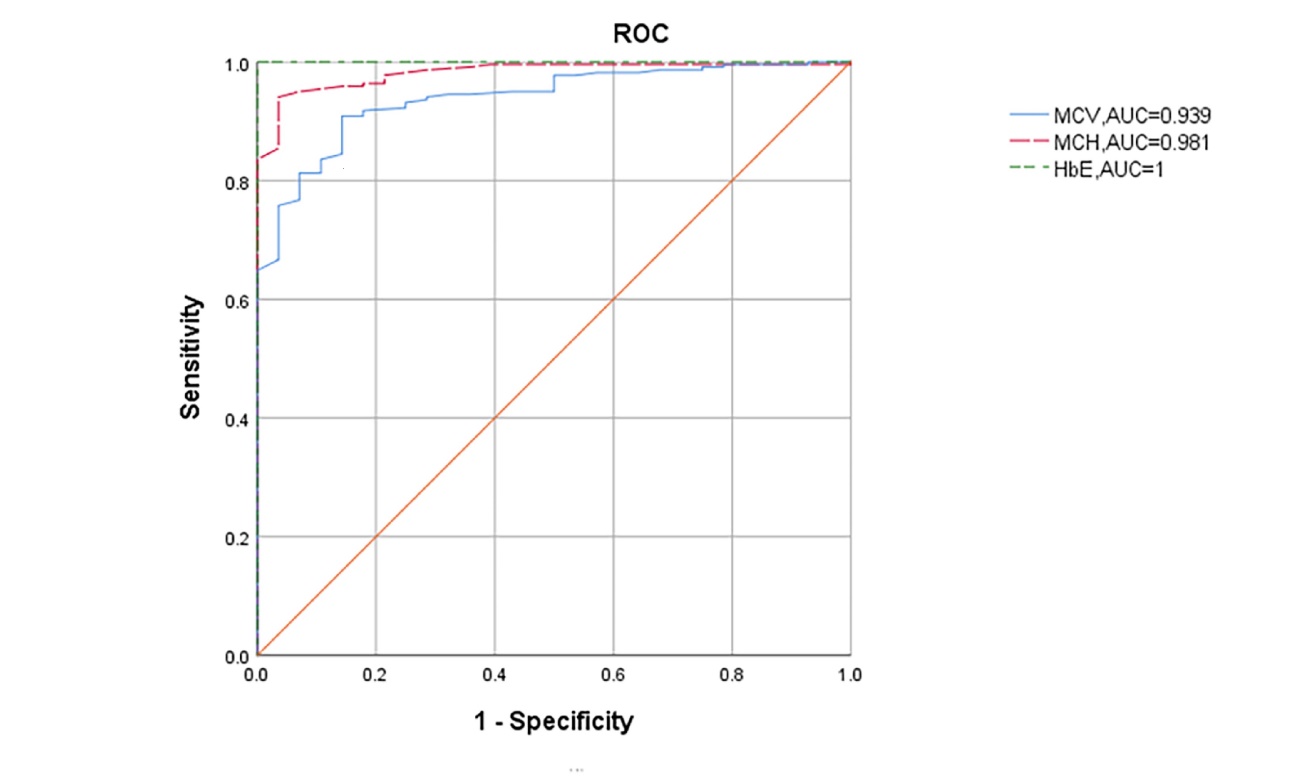


**Figure Legends**

**F I G U R E S1 (**a) MLPA result of the person with novel δβ thalassemia. (b) Based on the result about deletional point using NGS (position 5236000 to 5258000), we identified the deletion was 21410 bp length and found a insert fragment about 5bp.(chr 11:5236351-5257775, GRCh37/hg19).

**F I G U R E S2** The proband presented mild anemia. Her Hb was 144g/L, MCVwas 66.9fL, MCH was 21.0 pg, HbA2 was 1.4%. The result of gap-PCR was --^SEA^/--^SEA^ not consistent with hematological phenotypes**. (**a) Comparing α1 ,α2 globin gene sequences and the sequencing obtained using primers for HBA2 gene in our study. (b)MLPA result of the person with rare α^0^ thalassemia; the ratios for 24 probes showing heterozygote of South-East Asian (SEA)-type deletion accrording to Product Description SALSA® MLPA® probemix P140-C1 HBA (MRC-Holland).The ratios for probes only detecting HBA2 gene was nearly zero while the ratios for probes detecting the sequence in HBA1 were almost normal. (c)Schematic representation of the fusion gene between Z2 and Z1 box segments. Direct sequencing of three segments of Z2 and Z1 box. It demonstrated that the location of the fusion event happened in the first segment.(Z2 box: chr 16: 222427-222868; Z1 box: chr 16:226231-226272, GRCh37/hg19)

**F I G U R E S3** The ROC curve of Hb E 19.4% (CE), MCV 72.27 fL, and MCH 23.5 pg for detection of α^0^-thalassemia carriers in HbE heterozygotes.
