# Supplementary material for: Evaluation of intervention strategy of thalassemia for couples of childbearing ages in Centre of Southern China
Source: J Clin Lab Anal. 2021 Sep 7;35(10):e23990. doi: 10.1002/jcla.23990 (PMC8529143; doi:10.1002/jcla.23990)
Supplement: Supplementary file 2 — Table S1 [file JCLA-35-e23990-s002.docx]

**TABLE S1** Hematological data for abnormal Hb including compound thalassemia

| Type | Number | Hb(g/L) | MCV(fL) | MCH(pg) | HbA2  （%） | Hb variants  （%） |
| --- | --- | --- | --- | --- | --- | --- |
| Hb Q-Thailand（HBA1:c.223G>C） | 97 | 136.22±15.53 | 78.92±3.68 | 26.03±1.49 | 1.77±0.15 | 28.66±0.96 |
| Hb Q-Thailand, β^-28(A>G)^/β^N^ | 1 | 147.00 | 80.90 | 26.40 | 4.20 | 24.60 |
| Hb Q-Thailand, β^CD41-42(-CTTT)^/β^N^ | 2 | 142.00±8.48 | 64.50±2.82 | 21.35±0.21 | 4.65±0.07 | 16.7±0.7 |
| Hb Q-Thailand, β^IVS2-654(C>T)^/β^N^ | 1 | 143.00 | 65.50 | 20.20 | 4.20 | 14.20 |
| HbH-Q | 2 | 101.00，84.00 | 60.00, 54.30 | 17.40,17.80 | 0.00, 0.90 | 89.30，79.30 |
| Hb NewYork（HBB:c.341T>A） | 65 | 139.97±18.50 | 86.56±6.38 | 28.14±2.61 | 2.89±0.24 | 43.54±1.51 |
| Hb NewYork, β ^IVS2-654(C>T)^/β^N^ | 3 | 116.66±14.97 | 66.26±3.21 | 19.87±0.55 | 5.03±0.23 | 94.33±0.20 |
| Hb NewYork, β^-28(A>G)^/β^N^ | 1 | 121.00 | 74.60 | 23.70 | 6.20 | 79.20 |
| Hb NewYork, --^SEA^/αα | 12 | 114.00±7.97 | 67.31±2.01 | 20.65±0.90 | 2.85±0.13 | 36.27±1.18 |
| Hb NewYork, -α^3.7^/αα | 8 | 134.00±19.25 | 79.79±6.78 | 24.98±2.73 | 2.83±0.26 | 39.33±2.6 |
| Hb NewYork, -α^4.2^/αα | 4 | 126.50±12.79 | 81.15±3.72 | 26.48±0.89 | 2.93±0.24 | 40.03±0.82 |
| HbJ-Bangkok（HBB:c.170G>A） | 23 | 140.70±12.52 | 87.99±2.93 | 28.81±1.57 | 2.58±0.44 | 50.36±3.98 |
| HbJ-Bangkok , --^SEA^/αα | 7 | 125.00±12.17 | 70.17±3.70 | 21.74±1.04 | 1.98±0.40 | 49.64±2.14 |
| HbJ-Bangkok, β ^IVS2-654(C>T)^/β^N^ | 1 | 115.00 | 70.40 | 20.20 | 4.60 | 94.10 |
| HbJ-Bangkok, Hb Q-Thailand | 1 | 142.00 | 75.90 | 25.50 | 2.60 | Hb Q-Thailand:16.4%;HbJ-Bangkok:44.65； HbJ-Bangkok- Hb Q-Thailand：8.8% |
| Hb G-Taipei（HBB:c.68A>G） | 6 | 134.75±18.46 | 84.75±4.89 | 28.25±2.29 | 3.13±0.21 | 38.07±3.06 |
| Hb G-honolulu HBA2:c.91G>C | 1 | 77.30, | 88.10 | 29.00 | 1.90 | 23.60 |
| Hb G-honolulu, β^-28(A>G)^/ β^N^ | 2 | 113.00, 113.00 | 77.30, 70.03 | 24.60, 22.20 | 4.40, 4.50 | 18.00,18.80 |
| Hb G-honolulu, β^IVS2-654(C>T)^/β^N^ | 1 | 131.00 | 57.40 | 19.90 | 4.40 | 17.70 |
| Hb G-honolulu, β^CD17(A>T)^/ β^N^ | 1 | 127.00 | 57.90 | 18.60 | 4.50 | 18.20 |
| Hb S（HBB:c.20A>T） | 3 | 156.33±8.96 | 82.10 | 25.83±1.35 | 3.20±0.26 | 36.05±3.60 |
| Hb G-Siriraj（HBB:c.22G>A） | 3 | 157.5±16.16 | 83.2±6.16 | 28.45±0.80 | 3.20±0.94 | 31.50±3.47 |
| Hb G-Siriraj, -α^3.7^/αα | 1 | 157.00 | 76.90 | 26.30 | 3.20 | 20.30 |
| Hb Queens（HBA2: c.104T>G） | 1 | 146.00 | 91.80 | 29.90 | 2.30 | 17.20 |
| Hb Queens, β^CD41-42(-CTTT)^/β^N^ | 1 | 111.00 | 65.30 | 21.50 | 4.80 | 9.10 |
